# Determination of genetic effects of *ATF3* and *CDKN1A* genes on milk yield and compositions in Chinese Holstein population

**DOI:** 10.1186/s12863-017-0516-4

**Published:** 2017-05-19

**Authors:** Bo Han, Weijun Liang, Lin Liu, Yanhua Li, Dongxiao Sun

**Affiliations:** 1 Department of Animal Genetics, Breeding and Reproduction, College of Animal Science and Technology, Key Laboratory of Animal Genetics and Breeding of Ministry of Agriculture, National Engineering Laboratory of Animal Breeding, China Agricultural University, Beijing, 100193 China; 2 Beijing Dairy Cattle Center, Beijing, 100192 China

**Keywords:** Genetic effects, *ATF3*, *CDKN1A*, Milk production traits, Chinese Holstein

## Abstract

**Background:**

Our previous RNA-sequencing study revealed that the *ATF3* and *CDKN1A* genes were remarkably differentially expressed between the mammary glands of lactating Holstein cows with extremely high and low milk protein and fat percentage so that both of them were considered as candidates for milk composition. Herein, we further verified whether these genes have genetic effects on milk production traits in a Chinese Holstein cow population.

**Results:**

By re-sequencing the entire coding and regulatory regions, we identified four SNPs in 5’promoter region, two in exons, seven in 3′ un-translated region (UTR), and six in 3’flanking region of *ATF3* gene, and one SNP in exon 5, two in 3’UTR, and two in 3’flanking region of *CDKN1A* gene. Of these, only the SNP, c.271C > T (rs442346530), in exon 5 of *CDKN1A* gene was predicted to result in an amino acid replacement from arginine to tryptophan. Subsequent genotype-phenotype association analysis revealed that 19 SNPs in *ATF3* and 5 SNPs in *CDKN1A* were evidently associated with 305-days milk yield, fat yield, protein yield, or protein percentage (*P* = < 0.0001 ~ 0.0494). Whilst, no significant SNPs in *ATF3* gene were associated with fat percentage in both first and second lactations (*P* > 0.05), and only two SNPs of *CDKN1A* gene, c.271C > T (*P* = 0.0377) and c.*654C > T (*P* = 0.0144), were markedly associated with fat percentage in the first lactation. Further, linkage disequilibrium (LD) analyses were conducted among the identified SNPs in *ATF3* and/or *CDKN1A* genes to further confirm the association results. We also observed that the four SNPs, g.72834301C > A, g.72834229C > A, g.72833969A > G, and g.72833562G > T altered the specific transcription factor (TF) binding sites in *ATF3* promoter, and one SNP, c.271C > T, changed the CDKN1A protein secondary structure, suggesting they might be the promising potential functional mutations.

**Conclusion:**

Our findings first profiled the genetic effects of *ATF3* and *CDKN1A* genes for milk production traits in dairy cattle and will be available for marker-assisted breeding in dairy cattle.

**Electronic supplementary material:**

The online version of this article (doi:10.1186/s12863-017-0516-4) contains supplementary material, which is available to authorized users.

## Background

Milk production traits are the most important economic traits in dairy development, including milk yield, fat yield, protein yield, fat percentage and protein percentage [1]. As we know, most economic traits of livestock are quantitative traits, caused by minor genes. In the past several decades, quantitative trait locus (QTL) mapping, candidate gene analysis, and genome-wide association study (GWAS) have been used to identify the causal genes or mutations for milk production in dairy cattle [2–4]. However, only the causative mutations in *DGAT1*, *GHR*, and *ABCG2* gene, have been confirmed to date [5–7].

RNA sequencing (RNA-Seq) has become a comprehensive and accurate tool for analyzing the gene expression pattern [8]. Our previous RNA-Seq study has identified 31 differentially expressed genes between the mammary glands of lactating Chinese Holstein cows with the high and low milk protein percentage and fat percentage, of which, 17 genes are located within the known QTL regions for milk protein and fat traits and 21 contain or are near to the significant SNPs identified by previous GWAS, including *ATF3* and *CDKN1A* [9]. The *ATF3* gene (chr16: 72,820,025–72,832,974) were 1.9 Mb or 3.3 Mb close to the two SNPs, ARS-BFGL-NGS-70836 (chr16: 74,741,746) associated with milk protein percentage (*P* < 3.58E-07) and ARS-BFGL-NGS-85980 (chr16: 76,091,078) associated with fat percentage (*P* < 6.46E-07) and protein percentage (*P* < 8.33E-09) detected by a previous GWAS in dairy cattle [10]. *ATF3* (activating transcription factor 3) is a transcription factor belonging to the mammalian activation transcription factor/cAMP responsive element-binding (CREB) protein family [11]. Our GO and Ingenuity Pathway Analysis (IPA) also found that *ATF3* was involved in the lipid metabolism pathway, accumulation of glycoproteins, apoptosis, and regeneration of epithelial cells so that it is thought to be related to milk fat formation and development of mammary epithelial cells [9]. In addition, the *CDKN1A* gene (chr23: 10,560,498–10,568,780) is located within the known QTL regions (BTA23: 12.4 cM) that were confirmed to have large genetic effects on milk protein percentage [12]. *CDKN1A* (cyclin dependent kinase inhibitor 1A) encodes a 21-kD protein (p21) that is a cyclin-dependent kinase inhibitor, which is a rate-limiting regulator in the transition from G1 to S phase [13], and is also a key mediator of growth arrest that induced by the tumor suppressor protein p53 [14]. So far, there is no study revealing the associations of *ATF3* and *CDKN1A* genes with milk production in dairy cattle.

Based on our previous RNA-Seq study [9], the purpose of this study was to identify whether *ATF3* and *CDKN1A* genes have a genetic effect on milk production traits in dairy cows. Herein, we detected the SNPs in *ATF3* and *CDKN1A* genes, and analyzed the association between these polymorphisms with five milk production traits in a Chinese Holstein population. In addition, bioinformatics analysis was performed to profile the promoter activity and protein structure variation of *ATF3* and *CDKN1A* genes.

## Methods

### Animals and phenotypic data

A total of 1093 Chinese Holstein cows from 40 sire families were used in this study, and each sire had between 6 and 70 daughters with approximately 27 daughters on average per sire. These cows were from 22 dairy farms belonging to the Sanyuanlvhe Dairy Farming Center, where standard performance testing for dairy herd improvement (DHI) has been regularly conducted since 1999. Estimate breeding values (EBVs) for 305-days milk yield, fat yield, fat percentage, protein yield, and protein percentage during the first and second lactations were provided by the Beijing Dairy Cattle Center (http://www.bdcc.com.cn/). The descriptive statistics of phenotypic values for dairy production traits in two lactations were shown in Additional file 1. All protocols for sample collections of experimental individuals and phenotypic observations were approved by the Institutional Animal Care and Use Committee (IACUC) at China Agricultural University, and the permit number is DK996.

### DNA extraction

The whole blood samples of 1093 Chinese Holstein cows were extracted by TIANamp Blood DNA Kit (Tiangen, Beijing, China) according to the manufacturer’s instructions. The genomic DNA from semen samples of the sires were extracted using the standard salt-out procedures. The quantity and quality of the extracted DNA were respectively measured using a NanoDrop 2000 Spectrophotometer (Thermo Scientific, DE, USA) and by the gel electrophoresis.

### SNP identification and genotyping

We designed a total of 35 primers (Additional file 2) using Primer 3.0 (http://primer3.wi.mit.edu/) and Oligo 6.0 (Molecular Biology Insights, Inc., CO, USA) to amplify the entire coding region and 2000 bp of flanking sequences based on the genomic sequence of the bovine *ATF3* (GenBank accession no.: AC_000173.1) and *CDKN1A* (GenBank accession no.: AC_000180.1) genes, and the primers were synthesized by the Beijing Genomics Institute (BGI, Beijing, China). We randomly divided the DNA samples of the 40 sires into two equal groups, and diluted all the DNA samples into the concentration of 50 ng/μL. Subsequently, we constructed two DNA pools (50 ng/mL) as the templates for the PCR amplification. The amplifications were performed using the PCR system or the touch-down PCR (Additional file 2). The purified PCR products were directly sequenced by ABI3730XL DNA analyzer (Applied Biosystems, Foster City, CA, USA), and the sequences were compared by DNAMAN 6.0 (Lynnon Biosoft, USA) and NCBI-BLAST (https://blast.ncbi.nlm.nih.gov/Blast.cgi) to search potential SNPs. The identified SNPs were further individually genotyped for all the 1093 Chinese Holstein cows using the matrix-assisted laser desorption/ionization time of flight mass spectrometry (MALDI-TOF MS, Sequenom MassARRAY, Bioyong Technologies Inc. HK).

### Association analyses

Association analyses between SNP genotypes and/or haplotypes and the five milk traits were conducted by SAS 9.13 (SAS Institute Inc., Cary, NC, USA), based on the following animal model:

$$ \mathrm{Y}=\upmu +\mathrm{hys}+\mathrm{b}\times \mathrm{M}+\mathrm{G}+\mathrm{a}+\mathrm{e} $$

where, Y was the phenotypic values of each trait for the cows; μ was the overall mean; hys was the fixed effect of farm, year, and season; b was the regression coefficient of covariant M; M was the fixed effect of calving month; G was the SNP genotype or haplotype effect; a was the individual random additive genetic effect, distributed as $\mathrm{N}\left(0,\mathrm{A}{\updelta}_a^2\right)$ $\mathrm{N}\;\left({0,\mathrm{A}\updelta}_{\mathrm{a}}^2\right)$, with the additive genetic variance ${\updelta}_{\mathrm{a}}^2$; and e was the random residual, distributed as $\mathrm{N}\;\left({0,\mathrm{I}\updelta}_{\mathrm{e}}^2\right)$, with identity matrix I and residual error variance ${\updelta}_{\mathrm{e}}^2$. Bonferroni correction was performed for multiple testing, and the significant level of the multiple tests was equal to the raw *P* value divided by number of tests. Currently, we considered the statistically significant association from being null effect if a raw *P* value is less than 0.05/N, where N represents the number of SNP loci.

We calculated the additive effect (a), dominant effect (d), and substitution effect (α) using SAS 9.13, and the computational formula was as follows: $\mathrm{a}=\frac{\mathrm{AA}\hbox{-} \mathrm{BB}}{2};\mathrm{d}=\mathrm{AB}\hbox{-} \frac{\mathrm{AA}+\mathrm{BB}}{2};\upalpha =\mathrm{a}+\mathrm{d}\;\left(\mathrm{q}\hbox{-} \mathrm{p}\right)$. Where, AA, BB, and AB were the least squares mean of the milk production traits in the corresponding genotype.

### Haplotype analysis

The linkage disequilibrium (LD) extent between all SNPs and haplotype blocks were estimated using Haploview 4.2 (Broad Institute of MIT and Harvard, Cambridge, MA, USA).

### Bioinformatics analysis

To analysis the biological functions of the SNPs, we used the JASPAR database (http://jaspar.binf.ku.dk/cgi-bin/jaspar_db.pl?rm=browse&db=core&tax_group=vertebrates) to profile the genetic variants of the transcription factor binding sites (TFBSs) of the SNPs associated with milk production traits in the 5′ promoter region (relative score > 0.85). In addition, we utilized NPSA SOPMA SERVER (https://npsa-prabi.ibcp.fr/cgi-bin/npsa_automat.pl?page=/NPSA/npsa_sopma.html) to predict the variations of protein secondary structure caused by missense mutation in the coding regions, and the parameters were window width (17), similarity threshold (8), and number of states (4).

## Results

### SNPs identification

After sequencing the entire coding and up/downstream regions, we totally identified 19 and five SNPs for *ATF3* and *CDKN1A* genes, respectively, and all the identified SNPs have been reported in the NCBI database (Table 1). There were four SNPs in the 5’promoter region, two in exons, seven in the 3’UTR, and six in the 3’flanking region of *ATF3* gene. For *CDKN1A* gene, one SNP, c.271C > T, was located in the exon 5, c.*9C > G and c.*654C > T in the 3’UTR, and g.10569766 T > C and g.10569779 T > C in the 3’flanking region. Of these, only the SNP in exon 5 of *CDKN1A* gene (c.271C > T) was predicted to result in an amino acid replacement from arginine (CGG) to tryptophan (UGG), and the two SNPs of *ATF3* gene in the coding region (c.291G > A and c.489G > A) were synonymous mutations.

**Table 1:** Information about identified SNPs of *ATF3* and *CDKN1A* genes

Gene	Gene region	SNPs	Position (UMD 3.1)	Allele	Amino acid missence	GenBank no.	Origin
ATF3	5′ promoter region	g.72834301C > A	chr16:72,834,301	C/A		rs41634778	NCBI
		g.72834229C > A	chr16:72,834,229	C/A		rs41634777	NCBI
		g.72833969A > G	chr16:72,833,969	A/G		rs41823579	NCBI
		g.72833562G > T	chr16:72,833,562	G/T		rs41823578	NCBI
	exon 4	c.291G > A	chr16:72,822,913	G/A		rs209694892	NCBI
	exon 5	c.489G > A	chr16:72,821,334	G/A		rs207598277	NCBI
	3’UTR	c.*190A > G	chr16:72,821,087	A/G		rs136869959	NCBI
		c.*321G > C	chr16:72,820,956	G/C		rs135704995	NCBI
		c.*326A > G	chr16:72,820,951	A/G		rs137084971	NCBI
		c.*640G > A	chr16:72,820,637	G/A		rs210941907	NCBI
		c.*685G > C	chr16:72,820,592	G/C		rs209048412	NCBI
		c.*735 T > C	chr16:72,820,542	T/C		rs210174407	NCBI
		c.*1064G > A	chr16:72,820,213	G/A		rs211390316	NCBI
	3′ flanking region	g.72819977 T > C	chr16:72,819,977	T/C		rs208866610	NCBI
		g.72819850A > G	chr16:72,819,850	A/G		rs211185269	NCBI
		g.72818819A > G	chr16:72,818,819	A/G		rs41823568	NCBI
		g.72818818C > T	chr16:72,818,818	C/T		rs41823569	NCBI
		g.72818292 T > C	chr16:72,818,292	T/C		rs209896483	NCBI
		g.72818161 T > C	chr16:72,818,161	T/C		rs210138925	NCBI
CDKN1A	exon 5	c.271C > T	chr23:10,565,335	C/T	p.Arg91Trp	rs442346530	NCBI
	3’UTR	c.*9C > G	chr23:10,567,031	C/G		rs210197882	NCBI
		c.*654C > T	chr23:10,567,676	C/T		rs110284249	NCBI
	3’flanking region	g.10569766 T > C	chr23:10,569,766	T/C		rs133659402	NCBI
		g.10569779 T > C	chr23:10,569,779	T/C		rs110283961	NCBI

In addition, in this study, we performed the pooled sequencing method to identify the allelic variants across the entire coding and regulatory regions of *ATF3* and *CDKN1A* genes, and detected a total of 24 SNPs. However, this method has disadvantage that it may miss rare allelic variants due to sequencing cannot catch the very low fluorescent signal of the alleles with very low frequencies. Further, the allelic frequencies cannot be obtained merely by pooled sequencing, hence, we further genotyped 1093 cows and performed association analysis.

### Associates between SNPs and five milk traits

Associations between the total 24 SNPs of *ATF3* and *CDKN1A* genes and five milk production traits were presented in Tables 2 and 3. Using single-SNP association analysis, we found that 14, 16, 16 and 3 SNPs of *ATF3* gene were significantly associated with milk yield (*P* = < 0.0001 ~ 0.031), fat yield (*P* = 0.0002 ~ 0.0197), protein yield (*P* = < 0.0001 ~ 0.0331), and protein percentage (*P* = 0.0108 ~ 0.0494) in the first lactation, respectively. For example, the cows with allele C in g.72834301C > A showed higher milk yield, milk protein yield and fat yield than that with allele A **(**Table 2 **)**. Similarly, 13, 14, 11 and 8 SNPs were associated with milk yield (*P* = < 0.0001 ~ 0.0078), fat yield (*P* = < 0.0001 ~ 0.0395), protein yield (*P* = < 0.0001 ~ 0.0461), and protein percentage (*P* = 0.0003 ~ 0.0371) in the second lactation, respectively **(**Table 2). Of these, 13 SNPs of *ATF3* gene (g.72834301C > A, g.72834229C > A, g.72833969A > G, g.72833562G > T, c.291G > A, c.489G > A, c.*685G > C, c.*1064G > A, g.72819850A > G, g.72818819A > G, g.72818818C > T, g.72818292 T > C, and g.72818161 T > C) were identified significantly associated with at least one milk trait in both the first and second lactations. Four SNP, c.*190A > G, c.*326A > G, c.*640G > A, and g.72819977 T > C, were only found evidently associated with the milk traits in the first lactation, and the other two SNPs, c.*321G > C and c.*735 T > C, were markedly associated with at least one milk trait in the second lactation (Table 2), however, the allelic effects of these six SNPs showed almostly same directions between the first and second lactations although some associations did not reach statistical significance of 0.05 (Table 2). Interestingly, no significant SNPs were associated with fat percentage in both first and second lactations.

**Table 2:** Associations of 19 SNPs of *ATF3* gene with milk production traits in Chinese Holstein cattle during two lactations (LSM ± SE)

SNPs	Lactation	Genotype (No.)	Milk yield (kg)	Fat yield (kg)	Fat percentage (%)	Protein yield (kg)	Protein percentage (%)
g.72834301C > A	1	CC(222)	10,305 ± 73.52^a^	346.63 ± 3.19^Aa^	3.37 ± 0.03	306.89 ± 2.32^Aa^	2.98 ± 0.01
		CA(553)	10,339 ± 62^Aa^	343.59 ± 2.77^Aa^	3.34 ± 0.026	305.6 ± 2.01^Aa^	2.96 ± 0.008
		AA(299)	10,146 ± 68.79^Bb^	336.42 ± 3.01^B^	3.33 ± 0.028	300.12 ± 2.19^B^	2.96 ± 0.009
		*P*	0.001**	0.0002**	0.2065	0.0003**	0.0737
	2	CC(170)	10,707 ± 78.61^ab^	385.28 ± 3.39^ab^	3.61 ± 0.032	315.96 ± 2.47^Aa^	2.96 ± 0.011^Aa^
		CA(371)	10,785 ± 64.99^Aa^	390.15 ± 2.89^Aa^	3.63 ± 0.027	322.03 ± 2.1^B^	2.99 ± 0.009^B^
		AA(212)	10,567 ± 73.79^Bb^	379.59 ± 3.21^Bb^	3.59 ± 0.03	312.96 ± 2.34^Aa^	2.96 ± 0.01^Aa^
		*P*	0.0051**	0.0004**	0.2901	<.0001**	0.0005**
g.72834229C > A	1	CC(221)	10,318 ± 73	346.25 ± 3.17^Aa^	3.36 ± 0.03	306.34 ± 2.31^Aa^	2.97 ± 0.01^a^
		CA(553)	10,315 ± 61.81	341.19 ± 2.75^b^	3.32 ± 0.026	303.89 ± 2.01^ab^	2.95 ± 0.008^b^
		AA(299)	10,194 ± 68.95	337.13 ± 3.02^Bb^	3.31 ± 0.028	301.01 ± 2.2^Bb^	2.96 ± 0.009^ab^
		*P*	0.0523	0.003**	0.1234	0.0212*	0.0494*
	2	CC(169)	10,691 ± 78.17^a^	383.01 ± 3.37^a^	3.6 ± 0.032	314.66 ± 2.45^Aa^	2.95 ± 0.011^Aa^
		CA(369)	10,802 ± 64.49^Aa^	388.4 ± 2.86^Aa^	3.61 ± 0.027	321.87 ± 2.08^B^	2.99 ± 0.009^B^
		AA(211)	10,493 ± 73.62^Bb^	374.92 ± 3.19^Bb^	3.58 ± 0.03	309.72 ± 2.32^Ab^	2.96 ± 0.01^Aa^
		*P*	<.0001**	<.0001**	0.5118	<.0001**	0.001**
g.72833969A > G	1	GG(300)	10,147 ± 68.91^Aa^	333.48 ± 3.02^Aa^	3.3 ± 0.028	299.66 ± 2.2^A^	2.96 ± 0.009
		GA(551)	10,318 ± 61.59^Bb^	338.88 ± 2.74^b^	3.31 ± 0.025	304.12 ± 2^Ba^	2.95 ± 0.008
		AA(222)	10,273 ± 72.9^ab^	342.05 ± 3.16^Bb^	3.35 ± 0.03	304.89 ± 2.3^Ba^	2.97 ± 0.010
		*P*	0.0049**	0.0033**	0.1466	0.0057**	0.053
	2	GG(211)	10,466 ± 75.09^A^	372.1 ± 3.27^A^	3.56 ± 0.031	309.3 ± 2.38^Aa^	2.96 ± 0.010^Aa^
		GA(369)	10,793 ± 65.5^Ba^	388.2 ± 2.91^Ba^	3.61 ± 0.027	321.77 ± 2.12^B^	2.99 ± 0.009^B^
		AA(169)	10,700 ± 78.46^Ba^	382.28 ± 3.38^Bb^	3.59 ± 0.032	315.32 ± 2.46^Ab^	2.95 ± 0.011^Aa^
		*P*	<.0001**	<.0001**	0.1837	<.0001**	0.0015**
g.72833562G > T	1	TT(221)	10,340 ± 73.11	347.61 ± 3.17^Aa^	3.37 ± 0.03	306.84 ± 2.31^a^	2.97 ± 0.01
		TG(552)	10,360 ± 62.09	343.16 ± 2.77^a^	3.33 ± 0.026	305.18 ± 2.02^a^	2.95 ± 0.008
		GG(299)	10,234 ± 68.85	338.54 ± 3.02^Bb^	3.32 ± 0.028	301.92 ± 2.2^b^	2.95 ± 0.009
		*P*	0.0512	0.0029**	0.097	0.0275*	0.0779
	2	TT(169)	10,769 ± 78.92^Aa^	386.76 ± 3.41^Aa^	3.6 ± 0.032	316.78 ± 2.48^Aa^	2.95 ± 0.011^Aa^
		TG(370)	10,824 ± 64.37^Aa^	391.65 ± 2.85^Aa^	3.63 ± 0.027	322.38 ± 2.07^B^	2.98 ± 0.009^B^
		GG(210)	10,528 ± 74.13^B^	377.34 ± 3.22^B^	3.59 ± 0.03	310.91 ± 2.34^Ab^	2.96 ± 0.01^Aa^
		*P*	<.0001**	<.0001**	0.2651	<.0001**	0.0014**
c.291G > A	1	AA(31)	9757.9 ± 144.69^A^	326.63 ± 5.95^Aa^	3.37 ± 0.058	288.83 ± 4.34^A^	2.98 ± 0.021
		AG(345)	10,202 ± 66.08^Ba^	339.19 ± 2.91^b^	3.34 ± 0.027	301.61 ± 2.12^Ba^	2.96 ± 0.009
		GG(697)	10,268 ± 60.55^Ba^	343.04 ± 2.71^Bc^	3.35 ± 0.025	303.66 ± 1.98^Ba^	2.96 ± 0.008
		*P*	0.0007**	0.0034**	0.6488	0.0008**	0.752
	2	AA(25)	10,866 ± 165.07	400.94 ± 6.75^Aa^	3.68 ± 0.066	319.04 ± 4.93	2.94 ± 0.024
		AG(226)	10,557 ± 73.54	382.33 ± 3.2^Bb^	3.62 ± 0.03	313.83 ± 2.33	2.98 ± 0.01
		GG(503)	10,683 ± 61.69	385.82 ± 2.77^b^	3.62 ± 0.026	317.26 ± 2.01	2.98 ± 0.008
		*P*	0.0504	0.0172*	0.6436	0.147	0.3771
c.489G > A	1	AA(31)	9774.7 ± 144.35^A^	326.99 ± 5.93^Aa^	3.36 ± 0.058	289.58 ± 4.32^A^	2.98 ± 0.021
		AG(344)	10,239 ± 65.93^Ba^	338.76 ± 2.9^c^	3.32 ± 0.027	302.48 ± 2.11^Ba^	2.96 ± 0.009
		GG(709)	10,294 ± 60.82^Ba^	342.86 ± 2.72^Bb^	3.34 ± 0.025	304.28 ± 1.98^Ba^	2.96 ± 0.008
		*P*	0.0006**	0.0033**	0.4033	0.0009**	0.5871
	2	AA(25)	10,971 ± 164.92^ab^	399.91 ± 6.75^a^	3.63 ± 0.066	322.88 ± 4.92^ab^	2.94 ± 0.024
		AG(225)	10,657 ± 73.09^Aa^	386.05 ± 3.17^b^	3.61 ± 0.03	318.62 ± 2.31^a^	2.98 ± 0.01
		GG(510)	10,847 ± 60.46^Bb^	390.87 ± 2.71^ab^	3.6 ± 0.025	323.16 ± 1.97^b^	2.97 ± 0.008
		*P*	0.005**	0.0395*	0.8064	0.0461*	0.1971
c.*190A > G	1	AA(584)	10,178 ± 62.42^a^	338.65 ± 2.78^Aa^	3.35 ± 0.026	300.85 ± 2.02	2.96 ± 0.008
		AG(444)	10,217 ± 63.84^Aa^	338.37 ± 2.83^Aa^	3.33 ± 0.026	301.91 ± 2.06	2.96 ± 0.009
		GG(56)	9939.48 ± 113.17^Bb^	323.63 ± 4.7^B^	3.28 ± 0.046	294.8 ± 3.43	2.98 ± 0.016
		*P*	0.031*	0.0014**	0.2273	0.0705	0.6105
	2	AA(423)	10,792 ± 63.54	389.14 ± 2.82	3.61 ± 0.026	321.2 ± 2.05	2.98 ± 0.008
		AG(295)	10,671 ± 67.97	387.7 ± 2.98	3.63 ± 0.028	318.92 ± 2.17	2.99 ± 0.009
		GG(42)	10,736 ± 131.2	385.86 ± 5.42	3.58 ± 0.053	316.43 ± 3.95	2.94 ± 0.019
		*P*	0.1326	0.7237	0.5687	0.249	0.0664
c.*321G > C	1	CC(53)	10,076 ± 114.66	329.19 ± 4.76	3.29 ± 0.046	298.66 ± 3.47	2.97 ± 0.016
		CG(435)	10,277 ± 63.65	339.07 ± 2.82	3.32 ± 0.026	303.06 ± 2.05	2.96 ± 0.009
		GG(587)	10,217 ± 62.36	338.28 ± 2.78	3.33 ± 0.026	301.31 ± 2.02	2.96 ± 0.008
		*P*	0.1191	0.0698	0.5569	0.2362	0.583
	2	CC(41)	10,584 ± 131.99	380.38 ± 5.45	3.6 ± 0.053	311.06 ± 3.97	2.94 ± 0.019^a^
		CG(290)	10,690 ± 69.23	388.18 ± 3.04	3.64 ± 0.028	319.27 ± 2.21	2.99 ± 0.009^b^
		GG(424)	10,706 ± 64.21	385.69 ± 2.86	3.62 ± 0.026	317.31 ± 2.08	2.97 ± 0.009^ab^
		*P*	0.6383	0.2827	0.6702	0.0947	0.0371*
c.*326A > G	1	AA(587)	10,223 ± 62.03^Aa^	337.47 ± 2.77^Aa^	3.32 ± 0.026	301.41 ± 2.01^Aa^	2.96 ± 0.008
		AG(431)	10,295 ± 63.85^Aa^	338.53 ± 2.83^Aa^	3.31 ± 0.026	303.33 ± 2.06^Aa^	2.95 ± 0.009
		GG(57)	9904.58 ± 111.55^B^	322.04 ± 4.64^B^	3.28 ± 0.045	292.88 ± 3.38^B^	2.97 ± 0.016
		*P*	0.0008**	0.0004**	0.5042	0.0026**	0.4722
	2	AA(425)	10,789 ± 64.31	389.29 ± 2.86	3.62 ± 0.027	319.31 ± 2.08	2.97 ± 0.009
		AG(285)	10,706 ± 68.79	387.76 ± 3.02	3.63 ± 0.028	318.88 ± 2.2	2.98 ± 0.009
		GG(43)	10,572 ± 129.83	382.68 ± 5.36	3.62 ± 0.052	310.75 ± 3.91	2.95 ± 0.019
		*P*	0.1309	0.3978	0.9601	0.0689	0.0988
c.*640G > A	1	AA(31)	9805.53 ± 145.34^A^	328.47 ± 5.97^Aa^	3.36 ± 0.058	290.54 ± 4.36^A^	2.97 ± 0.021
		AG(340)	10,228 ± 66.3^Ba^	341.02 ± 2.92^c^	3.34 ± 0.027	302.39 ± 2.13^Ba^	2.95 ± 0.009
		GG(709)	10,299 ± 60.44^Ba^	345.29 ± 2.7^Bb^	3.36 ± 0.025	304.73 ± 1.97^Ba^	2.96 ± 0.008
		*P*	0.0009**	0.0018**	0.5681	0.0009**	0.655
	2	AA(25)	10,910 ± 164.71	396.49 ± 6.74	3.63 ± 0.066	2.93 ± 0.024	2.93 ± 0.024
		AG(221)	10,653 ± 73.17	381.19 ± 3.18	3.58 ± 0.03	2.97 ± 0.01	2.97 ± 0.01
		GG(510)	10,763 ± 61.24	384.41 ± 2.75	3.58 ± 0.025	2.97 ± 0.008	2.97 ± 0.008
		*P*	0.107	0.0533	0.7617	0.2326	0.2326
c.*685G > C	1	CC(30)	9978.84 ± 146.26	328.2 ± 6	3.32 ± 0.059	294.37 ± 4.38^a^	2.96 ± 0.021
		CG(337)	10,272 ± 66.72	336.86 ± 2.94	3.3 ± 0.027	303.87 ± 2.14^b^	2.96 ± 0.009
		GG(713)	10,302 ± 60.16	339.63 ± 2.69	3.31 ± 0.025	304.85 ± 1.96^b^	2.96 ± 0.008
		*P*	0.0617	0.0588	0.6272	0.0331*	0.9781
	2	CC(25)	11,131 ± 164.65^Aa^	398.09 ± 6.74^a^	3.57 ± 0.066	323.53 ± 4.92^ab^	2.92 ± 0.024
		CG(221)	10,600 ± 73.44^B^	383.03 ± 3.19^b^	3.61 ± 0.03	314.94 ± 2.33^a^	2.97 ± 0.01
		GG(511)	10,774 ± 61.52^Ab^	388.76 ± 2.77^a^	3.61 ± 0.025	319.05 ± 2.01^b^	2.97 ± 0.008
		*P*	0.0008**	0.0168*	0.8299	0.0384*	0.05
c.*735 T > C	1	CC(30)	10,031 ± 146.66	330.74 ± 6.02	3.32 ± 0.059	296.27 ± 4.39	2.96 ± 0.021
		CT(338)	10,256 ± 66.98	337.13 ± 2.95	3.3 ± 0.028	303.04 ± 2.15	2.96 ± 0.009
		TT(712)	10,306 ± 60.38	339.99 ± 2.7	3.32 ± 0.025	304.31 ± 1.97	2.96 ± 0.008
		*P*	0.1031	0.1125	0.7774	0.1133	0.9598
	2	CC(25)	11,178 ± 166.12^Aa^	397.7 ± 6.82^a^	3.56 ± 0.067	326.05 ± 4.97^a^	2.92 ± 0.024
		CT(222)	10,608 ± 73.59^B^	383.22 ± 3.2^b^	3.61 ± 0.03	315.95 ± 2.33^b^	2.98 ± 0.01
		TT(511)	10,791 ± 61.3^Ab^	388.01 ± 2.75^ab^	3.6 ± 0.025	320.43 ± 2^a^	2.97 ± 0.008
		*P*	0.0003**	0.0358*	0.7454	0.0166*	0.0571
c.*1064G > A	1	AA(30)	9789.35 ± 145.71^A^	324.33 ± 5.97^Aa^	3.34 ± 0.058	290.87 ± 4.35^A^	2.98 ± 0.021
		AG(346)	10,225 ± 66.48^Ba^	336.45 ± 2.93^b^	3.31 ± 0.027	302.73 ± 2.13^Ba^	2.96 ± 0.009
		GG(699)	10,290 ± 60.6^Ba^	340.29 ± 2.71^Bb^	3.33 ± 0.025	304.73 ± 1.97^Ba^	2.96 ± 0.008
		*P*	0.0011**	0.0046**	0.6409	0.0019**	0.5754
	2	AA(24)	11,065 ± 169.52^a^	411.69 ± 6.93^A^	3.7 ± 0.068	324.69 ± 5.05	2.94 ± 0.024
		AG(227)	10,639 ± 72.64^b^	386.11 ± 3.16^Ba^	3.62 ± 0.03	317.56 ± 2.3	2.98 ± 0.01
		GG(501)	10,797 ± 61.59^a^	391.84 ± 2.76^Bb^	3.63 ± 0.025	321.28 ± 2.01	2.98 ± 0.008
		*P*	0.0062**	0.0004**	0.5116	0.0823	0.1711
g.72819977 T > C	1	TT(709)	10,252 ± 60.58^Aa^	341.13 ± 2.71^Aa^	3.34 ± 0.025	303.11 ± 1.97^Aa^	2.96 ± 0.008
		TC(345)	10,190 ± 66.68^Aa^	336.44 ± 2.94^b^	3.32 ± 0.027	301.02 ± 2.14^a^	2.96 ± 0.009
		CC(31)	9810.39 ± 144.46^B^	326.61 ± 5.93^Bb^	3.35 ± 0.058	290.6 ± 4.33^Bb^	2.97 ± 0.021
		*P*	0.0037**	0.0034**	0.391	0.0044**	0.6802
	2	TT(510)	10,795 ± 60.71	388.17 ± 2.72	3.6 ± 0.025	321.11 ± 1.98	2.97 ± 0.008
		TC(225)	10,673 ± 72.24	385.16 ± 3.13	3.6 ± 0.03	318.52 ± 2.28	2.98 ± 0.01
		CC(25)	10,953 ± 165.5	399.73 ± 6.77	3.64 ± 0.066	321.39 ± 4.94	2.94 ± 0.024
		*P*	0.0686	0.0736	0.777	0.3541	0.2628
g.72819850A > G	1	GG(31)	9737.41 ± 143.71^A^	322.7 ± 5.9^Aa^	3.34 ± 0.058	287.79 ± 4.3^A^	2.97 ± 0.021
		GA(342)	10,221 ± 66.54^Ba^	336.58 ± 2.93^b^	3.3 ± 0.027	301.93 ± 2.13^Ba^	2.95 ± 0.009
		AA(706)	10,282 ± 60.43^Ba^	340.2 ± 2.7^Bb^	3.32 ± 0.025	303.63 ± 1.97^Ba^	2.95 ± 0.008
		*P*	0.0003**	0.0021**	0.6324	0.0003**	0.7239
	2	GG(25)	10,948 ± 164.92^b^	404.59 ± 6.75^Aa^	3.69 ± 0.066	321.58 ± 4.92^ab^	2.94 ± 0.024
		GA(224)	10,546 ± 73.43^Aa^	381.72 ± 3.19^B^	3.62 ± 0.03	313.96 ± 2.32^Aa^	2.98 ± 0.01
		AA(508)	10,773 ± 61.24^Bb^	388.75 ± 2.75^Ab^	3.61 ± 0.025	320.15 ± 2^Bb^	2.98 ± 0.008
		*P*	0.0005**	0.0004**	0.5079	0.003**	0.2881
g.72818819A > G	1	GG(305)	10,154 ± 68.15^Aa^	336.43 ± 2.98^A^	3.33 ± 0.028	299.24 ± 2.17^A^	2.95 ± 0.009^a^
		GA(557)	10,314 ± 61.4^Bb^	341.97 ± 2.74^Ba^	3.33 ± 0.025	303.62 ± 1.99^Ba^	2.95 ± 0.008^Aa^
		AA(218)	10,301 ± 73.25^b^	346.69 ± 3.17^Bb^	3.38 ± 0.03	305.87 ± 2.31^Ba^	2.97 ± 0.01^Bb^
		*P*	0.0068**	0.0004**	0.1048	0.0012**	0.0108*
	2	GG(216)	10,481 ± 73.65^A^	374.35 ± 3.2^A^	3.57 ± 0.03	309.56 ± 2.33^A^	2.96 ± 0.01^Aa^
		GA(373)	10,845 ± 64.47^Ba^	391.01 ± 2.86^Ba^	3.61 ± 0.027	323.76 ± 2.08^B^	2.99 ± 0.009^B^
		AA(167)	10,778 ± 79.59^Ba^	384.6 ± 3.44^Bb^	3.57 ± 0.033	317.22 ± 2.51^C^	2.95 ± 0.011^Aa^
		*P*	<.0001**	<.0001**	0.1906	<.0001**	0.0003**
g.72818818C > T	1	TT(305)	10,224 ± 68.77^Aa^	337.97 ± 3.01^A^	3.31 ± 0.028	301.6 ± 2.19^A^	2.95 ± 0.009^ab^
		TC(555)	10,382 ± 61.58^Bb^	344.22 ± 2.75^Ba^	3.33 ± 0.025	305.98 ± 2^Ba^	2.95 ± 0.008^a^
		CC(221)	10,330 ± 72.84^ab^	346.52 ± 3.16^Ba^	3.36 ± 0.03	306.65 ± 2.3^Ba^	2.97 ± 0.01^b^
		*P*	0.0106*	0.0019**	0.1484	0.0075**	0.0355*
	2	TT(216)	10,466 ± 74^A^	374.26 ± 3.22^A^	3.58 ± 0.03	308.75 ± 2.34^A^	2.96 ± 0.01^Aa^
		TC(372)	10,767 ± 64.62^Ba^	389.23 ± 2.87^Ba^	3.63 ± 0.027	320.3 ± 2.09^Ba^	2.99 ± 0.009^B^
		CC(169)	10,737 ± 78.69^Ba^	385.23 ± 3.4^Ba^	3.6 ± 0.032	315.35 ± 2.48^Bb^	2.95 ± 0.011^Aa^
		*P*	<.0001**	<.0001**	0.1591	<.0001**	0.0006**
g.72818292 T > C	1	TT(709)	10,300 ± 60.49^Aa^	341.5 ± 2.71^a^	3.33 ± 0.025	304.06 ± 1.97^a^	2.96 ± 0.008
		TC(336)	10,249 ± 66.63^a^	337.35 ± 2.93^b^	3.31 ± 0.027	302.39 ± 2.13^a^	2.95 ± 0.009
		CC(33)	9929.1 ± 140.09^Bb^	330.35 ± 5.74^b^	3.34 ± 0.056	294.03 ± 4.19^b^	2.97 ± 0.02
		*P*	0.0156*	0.0197*	0.453	0.0253*	0.632
	2	TT(509)	10,683 ± 61.62^c^	382.44 ± 2.77^Aa^	3.6 ± 0.026	316.43 ± 2.02^a^	2.97 ± 0.008
		TC(219)	10,522 ± 72.71^Aa^	377.12 ± 3.15^Ab^	3.6 ± 0.03	312.89 ± 2.3^Aa^	2.98 ± 0.01
		CC(27)	11,074 ± 159.67^Bb^	405.34 ± 6.54^B^	3.64 ± 0.064	326.97 ± 4.77^Bb^	2.95 ± 0.023
		*P*	0.0006**	<.0001**	0.7811	0.0057**	0.4872
g.72818161 T > C	1	TT(711)	10,326 ± 60.82^Aa^	343.62 ± 2.72^A^	3.34 ± 0.025	305.45 ± 1.98^Aa^	2.96 ± 0.008
		TC(322)	10,206 ± 67.05^Ab^	338.41 ± 2.95^Ba^	3.32 ± 0.028	302.22 ± 2.15^Ab^	2.96 ± 0.009
		CC(30)	9725.31 ± 145.87^B^	325.45 ± 5.98^Bb^	3.36 ± 0.059	287.93 ± 4.36^B^	2.97 ± 0.021
		*P*	<.0001**	0.0004**	0.7091	<.0001**	0.6543
	2	TT(511)	10,714 ± 61.04^Aa^	383.57 ± 2.74^a^	3.59 ± 0.025	317.97 ± 1.99	2.97 ± 0.008^ab^
		TC(208)	10,546 ± 73.84^Bb^	378.91 ± 3.19^Aa^	3.6 ± 0.03	315.19 ± 2.33	2.99 ± 0.01^a^
		CC(24)	10,933 ± 168.72^a^	397.23 ± 6.9^Bb^	3.63 ± 0.068	319.26 ± 5.03	2.93 ± 0.024^b^
		*P*	0.0078**	0.014*	0.7997	0.2957	0.0239*

^*^ indicates *P* < 0.05; ^**^ indicates *P* < 0.01; ^a, b, c^ within the same column with different superscripts means *P* < 0.05; ^A, B, C^ within the same column with different superscripts means *P* < 0.01.

**Table 3:** Associations of 5 SNPs of *CDKN1A* gene with milk production traits in Chinese Holstein cattle during two lactations (LSM ± SE)

SNPs	Lactation	Genotype (No.)	Milk yield (kg)	Fat yield (kg)	Fat percentage (%)	Protein yield (kg)	Protein percentage (%)
c.271C > T	1	CC(582)	10,249 ± 62.43^a^	339.12 ± 2.79	3.32 ± 0.026^ab^	302.37 ± 2.03	2.95 ± 0.008
		CT(412)	10,212 ± 65.4^Aa^	341.67 ± 2.89	3.36 ± 0.027^a^	301.44 ± 2.11	2.96 ± 0.009
		TT(76)	10,478 ± 99.65^Bb^	340.05 ± 4.18	3.27 ± 0.04^b^	308.04 ± 3.05	2.95 ± 0.014
		*P*	0.0162*	0.414	0.0377*	0.0522	0.8062
	2	CC(394)	10,564 ± 64.48^Aa^	378.99 ± 2.86^Aa^	3.6 ± 0.027	313.03 ± 2.08^A^	2.97 ± 0.009^Aa^
		CT(302)	10,772 ± 67.72^Bb^	387.31 ± 2.98^Bb^	3.6 ± 0.028	319.25 ± 2.17^Ba^	2.97 ± 0.009^Aa^
		TT(54)	10,819 ± 119.09^b^	388.01 ± 4.94^ab^	3.61 ± 0.048	327.12 ± 3.6^Bb^	3.03 ± 0.017^B^
		*P*	0.0012**	0.0018**	0.9967	<.0001**	0.0012**
c.*9C > G	1	CC(593)	10,229 ± 61.78^a^	338.35 ± 2.76	3.33 ± 0.026	301.99 ± 2.01	2.96 ± 0.008
		CG(415)	10,173 ± 64.52^Aa^	339.45 ± 2.85	3.35 ± 0.027	300.37 ± 2.08	2.96 ± 0.009
		GG(76)	10,428 ± 99.99^Bb^	338.21 ± 4.19	3.28 ± 0.04	306.56 ± 3.06	2.95 ± 0.014
		*P*	0.0193*	0.8322	0.0956	0.0613	0.8896
	2	CC(401)	10,603 ± 64.7^A^	380.64 ± 2.87^Aa^	3.6 ± 0.027	314.09 ± 2.09^A^	2.97 ± 0.009^Aa^
		CG(305)	10,811 ± 67.06^Ba^	389.97 ± 2.96^Bb^	3.61 ± 0.028	320.55 ± 2.15^B^	2.97 ± 0.009^Aa^
		GG(54)	10,922 ± 117.94^Ba^	391.5 ± 4.89^b^	3.59 ± 0.048	329.78 ± 3.56^C^	3.02 ± 0.017^B^
		*P*	0.0004**	0.0003**	0.874	<.0001**	0.0055**
c.*654C > T	1	CC(497)	10,276 ± 62.96	337.95 ± 2.8	3.3 ± 0.026^Aa^	303.16 ± 2.04	2.95 ± 0.008
		CT(479)	10,213 ± 63.44	340.69 ± 2.82	3.35 ± 0.026^Bb^	301.57 ± 2.05	2.96 ± 0.008
		TT(107)	10,298 ± 90.26	338.05 ± 3.83	3.29 ± 0.037^ab^	303.44 ± 2.79	2.95 ± 0.013
		*P*	0.3235	0.3355	0.0144*	0.4693	0.7739
	2	CC(350)	10,862 ± 65.32	388.92 ± 2.89	3.58 ± 0.027	323.75 ± 2.11^Aa^	2.98 ± 0.009
		CT(330)	10,755 ± 67.61	389.17 ± 2.98	3.61 ± 0.028	319.07 ± 2.17^Bb^	2.96 ± 0.009
		TT(79)	10,756 ± 102.96	384.73 ± 4.33	3.58 ± 0.042	316.84 ± 3.16^b^	2.95 ± 0.014
		*P*	0.1847	0.529	0.5	0.0092**	0.0922
g.10569766 T > C	1	CC(79)	10,394 ± 98.78	334.84 ± 4.14	3.26 ± 0.04	304.06 ± 3.02	2.94 ± 0.014
		CT(427)	10,192 ± 65.04	336.77 ± 2.88	3.32 ± 0.027	299.51 ± 2.09	2.95 ± 0.009
		TT(564)	10,191 ± 62.48	335.13 ± 2.79	3.31 ± 0.026	299.53 ± 2.03	2.95 ± 0.008
		*P*	0.0672	0.6615	0.1899	0.2059	0.878
	2	CC(60)	10,803 ± 113.4^a^	386.17 ± 4.72^ab^	3.59 ± 0.046	326.9 ± 3.44^Aa^	3.03 ± 0.016^A^
		CT(309)	10,790 ± 67.29^Aa^	388.76 ± 2.96^Aa^	3.61 ± 0.028	320.1 ± 2.16^Ab^	2.97 ± 0.009^Ba^
		TT(380)	10,560 ± 65.37^Bb^	379.99 ± 2.9^Bb^	3.61 ± 0.027	313.48 ± 2.11^B^	2.98 ± 0.009^Ba^
		*P*	0.0004**	0.0015**	0.8386	<.0001**	0.0009**
g.10569779 T > C	1	CC(559)	10,286 ± 61.82	339.28 ± 2.76	3.32 ± 0.026	303.96 ± 2.01	2.96 ± 0.008
		CT(434)	10,251 ± 65.2	339.93 ± 2.89	3.33 ± 0.027	302.21 ± 2.1	2.95 ± 0.009
		TT(87)	10,353 ± 95.78	337.22 ± 4.03	3.28 ± 0.039	305.05 ± 2.94	2.96 ± 0.013
		*P*	0.4605	0.7357	0.4159	0.3306	0.7423
	2	CC(374)	10,598 ± 64.94^Aa^	382.05 ± 2.88^Aa^	3.62 ± 0.027	314.19 ± 2.09^A^	2.97 ± 0.009^Aa^
		CT(316)	10,799 ± 66.94^Bb^	388.48 ± 2.95^Bb^	3.61 ± 0.028	319.81 ± 2.15^Ba^	2.97 ± 0.009^Aa^
		TT(65)	10,726 ± 108.83^ab^	381.24 ± 4.54^ab^	3.57 ± 0.044	323.07 ± 3.31^Ba^	3.02 ± 0.015^B^
		*P*	0.0041**	0.0197*	0.6029	0.001**	0.0035**

^*^ indicates *P* < 0.05; ^**^ indicates *P* < 0.01; ^a, b, c^ within the same column with different superscripts means *P* < 0.05; ^A, B, C^ within the same column with different superscripts means *P* < 0.01.

Regarding *CDKN1A* gene, it showed that c.271C > T (*P* = 0.0162) and c.*9C > G (*P* = 0.0193) were significantly associated with 305-days milk yield, and c.271C > T (*P* = 0.0377) and c.*654C > T (*P* = 0.0144) were markedly associated with fat percentage in the first lactation. Four identified SNPs of *CDKN1A* gene, c.271C > T, c.*9C > G, g.10569766 T > C, and g.10569779 T > C, were significantly associated with 305-days milk yield, fat yield, protein yield, and protein percentage (*P* = < 0.0001 ~ 0.0197), and c.*654C > T was only evidently associated with protein yield (*P* = 0.0092) in the second lactation (Table 3). Additionally, three SNPs of *CDKN1A* gene, c.271C > T, c.*9C > G, and c.*654C > T, were both significantly associated with milk traits in both first (*P* = 0.0144 ~ 0.0377) and second (*P* = < 0.0001 ~ 0.0197) lactations. The g.10569766 T > C and g.10569779 T > C were merely associated with milk traits in the second lactation (*P* = < 0.0001 ~ 0.0197), but the allelic effects of them were almost in the same direction between two lactations. Taken together, the identified SNPs of *CDKN1A* gene were mainly associated with the milk yield, fat yield, protein yield, and protein percentage in the second lactation of Chinese Holstein cattle in this study (Table 3).

The allele additive, dominant, and substitution effects of 19 SNPs identified in *ATF3* gene were mainly significantly associated with milk yield, fat yield, and protein yield (*P* < 0.05), and the results in the first lactation were slightly different from that in the second lactation; nevertheless, it is interesting that only two SNPs, g.72833562G > T and g.72818819A > G, were evidently associated with fat percentage in the first lactation (*P* < 0.05) (Additional file 3). Nevertheless, the allele additive, dominant, and substitution effects of 5 SNPs of *CDKN1A* gene were mainly associated with milk yield, protein yield, and protein percentage (*P* < 0.05) (Additional file 4).

### Associations between haplotypes and five milk traits

**Fig. 1:**
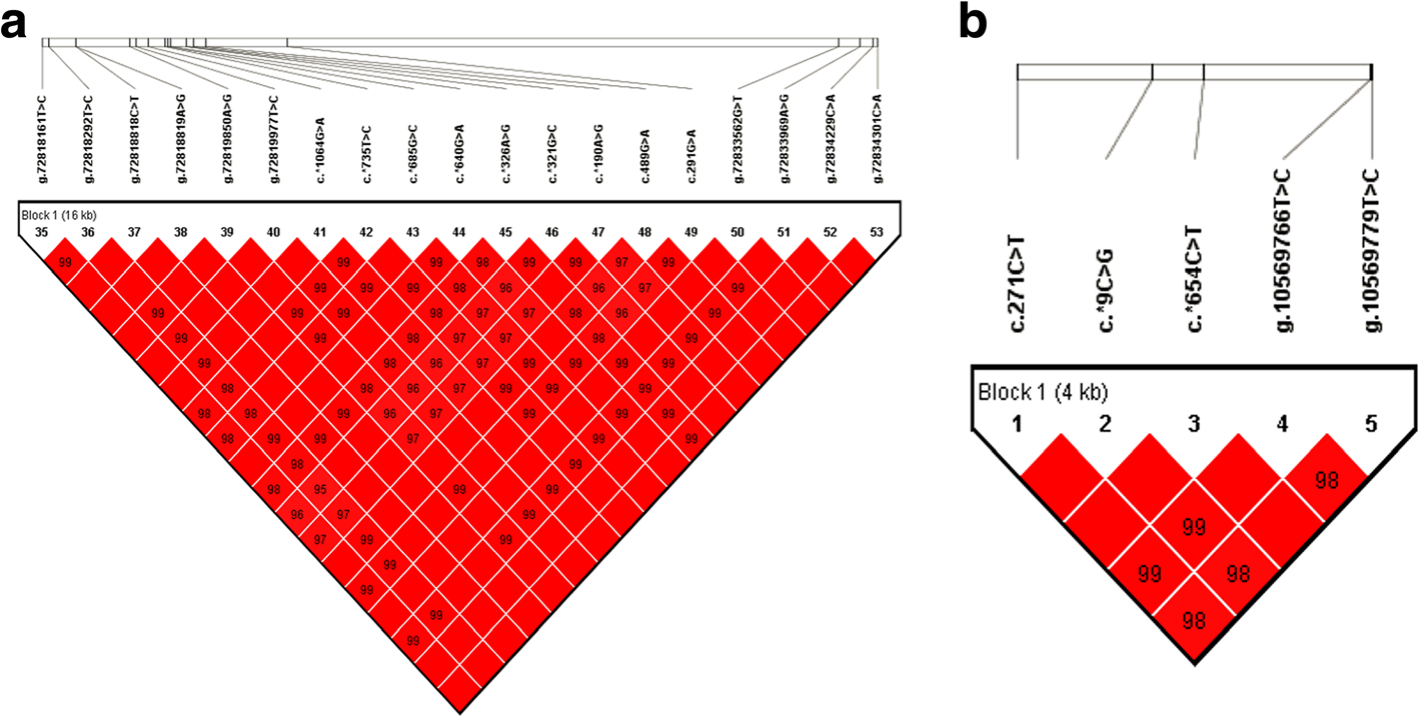
Linkage disequilibrium estimated between SNPs in *ATF3* (**a**) and *CDKN1A* (**b**) genes. The values in *boxes* are pair-wise SNP correlations (D’)

The pair-wise D’ measures indicated that all 19 SNPs in *ATF3* gene were highly linked (D’ > 0.95), and one haplotype block comprising the 19 SNPs was inferred (Fig. 1a), in which four haplotypes were formed. The frequency of the four haplotypes, H1 (AAGTTAGTGGAGAGGATGG), H2 (AAACTAGTGGAGAGGCCTT), H3 (GGACCGACCAGCGAACCTT), and H4 (AAACTAGTGGGCGGGCCTT), were 46.3%, 28.0%, 17.7%, and 7.2%, respectively. Haplotype-based association analysis showed that the six haplotype combinations were all significantly associated with 305-days milk yield, fat yield, protein yield, and protein percentage in the first lactation (*P* = 0.0019 ~ 0.0398), and evidently associated with 305-days milk yield, fat yield, and protein yield in the second lactation (*P* < 0.0001; Table 4).

**Table 4:** Haplotypes analysis of *ATF3* gene (LSM ± SE)

Lactation	Haplotype combination (No.)	Milk yield (kg)	Fat yield (kg)	Fat percentage (%)	Protein yield (kg)	Protein percentage (%)
1	H1H1 (220)	10,293 ± 73.81^ab^	344.45 ± 3.21^Aa^	3.36 ± 0.03	305.64 ± 2.34^ABa^	2.97 ± 0.01^Aa^
	H1H2 (286)	10,256 ± 70.59^abc^	337.6 ± 3.1^bc^	3.31 ± 0.029	301.1 ± 2.25^bc^	2.94 ± 0.01^Bb^
	H1H3 (184)	10,355 ± 77.21^Aab^	343.03 ± 3.34^Aab^	3.33 ± 0.032	305.97 ± 2.43^Aa^	2.96 ± 0.011^ab^
	H1H4 (84)	10,406 ± 100.1^Aa^	340.62 ± 4.21^abc^	3.29 ± 0.041	305.61 ± 3.07^ab^	2.95 ± 0.014^b^
	H2H2 (79)	10,165 ± 100.53^bc^	336.09 ± 4.22^bc^	3.32 ± 0.041	298.33 ± 3.08^BCc^	2.94 ± 0.014^b^
	H2H3 (125)	10,119 ± 86.34^Bc^	332.39 ± 3.69^Bc^	3.3 ± 0.035	297.98 ± 2.69^Cc^	2.95 ± 0.012^ab^
	*P*	0.0398*	0.0038**	0.3088	0.0019**	0.0334*
2	H1H1 (161)	10,740 ± 79.51^Aab^	389.62 ± 3.42^Aa^	3.64 ± 0.03	317.62 ± 2.49^Ab^	2.96 ± 0.011
	H1H2 (195)	10,813 ± 76.98^Aa^	389.73 ± 3.34^Aa^	3.62 ± 0.03	322.02 ± 2.43^Aab^	2.98 ± 0.011
	H1H3 (113)	10,625 ± 90.3^b^	390.54 ± 3.86^Aa^	3.68 ± 0.04	318.51 ± 2.81^Aab^	2.99 ± 0.013
	H1H4 (56)	10,887 ± 116.86^Aa^	392.75 ± 4.86^Aa^	3.61 ± 0.05	325.59 ± 3.54^Aa^	2.99 ± 0.017
	H2H2 (52)	10,293 ± 122.84^Bc^	369.21 ± 5.1^Bb^	3.6 ± 0.05	304.19 ± 3.72^Bc^	2.95 ± 0.017
	H2H3 (84)	10,405 ± 99.79^Bc^	374.45 ± 4.2^Bb^	3.6 ± 0.04	308.09 ± 3.06^Bc^	2.96 ± 0.014
	*P*	<.0001**	<.0001**	0.4721	<.0001**	0.0743

*H* means haplotype; H1: AAGTTAGTGGAGAGGATGG, H2: AAACTAGTGGAGAGGCCTT, H3: GGACCGACCAGCGAACCTT, and H4: AAACTAGTGGGCGGGCCTT; ^*^ indicates *P* < 0.05; ^**^ indicates *P* < 0.01; ^a, b, c^ within the same column with different superscripts means *P* < 0.05; ^A, B, C^ within the same column with different superscripts means *P* < 0.01.

Regarding the *CDKN1A* haplotypes, one block was inferred (D’ > 0.98) as shown in Fig. 1b, consisting of H1 (CCCTC), H2 (CCTTC), H3 (TGCCT), and H4 (CCCCT) with frequency of 39.9%, 31.7%, 25.7%, and 1.5%, respectively. The associations (Table 5) were indicated that the haplotype combinations were markedly associated with fat percentage (*P* = 0.044) and protein percentage (*P* = 0.0351) in the first lactation, and remarkably associated with 305-days milk yield, fat yield, protein yield, and protein percentage in the second lactation (*P* = < 0.0001 ~ 0.0163).

**Table 5:** Haplotypes analysis of *CDKN1A* gene (LSM ± SE)

Lactation	Haplotype combination (No.)	Milk yield (kg)	Fat yield (kg)	Fat percentage (%)	Protein yield (kg)	Protein percentage (%)
1	H1H1 (175)	10,246 ± 78.7	337.02 ± 3.4	3.3 ± 0.032^ab^	301.01 ± 2.47	2.94 ± 0.011^Bc^
	H1H2 (279)	10,190 ± 69.46	339.81 ± 3.04	3.35 ± 0.029^a^	302.54 ± 2.22	2.97 ± 0.009^Aa^
	H1H3 (222)	10,179 ± 74.12	338.05 ± 3.22	3.33 ± 0.03^ab^	301.48 ± 2.35	2.97 ± 0.01^ab^
	H2H2 (107)	10,250 ± 89.88	337.17 ± 3.81	3.3 ± 0.036^ab^	302.85 ± 2.78	2.96 ± 0.013^abc^
	H2H3 (179)	10,140 ± 77.68	339.79 ± 3.35	3.36 ± 0.032^Aa^	298.23 ± 2.44	2.95 ± 0.011^bc^
	H3H3 (75)	10,418 ± 100.99	336.31 ± 4.24	3.25 ± 0.041^Bb^	306.5 ± 3.09	2.95 ± 0.014^abc^
	*P*	0.1065	0.8508	0.044*	0.0939	0.0351*
2	H1H1 (114)	10,555 ± 90.95^Bc^	378.57 ± 3.87^Cc^	3.59 ± 0.037	314.92 ± 2.82^Bb^	2.98 ± 0.013^ABb^
	H1H2 (174)	10,623 ± 79.4^Bc^	387.32 ± 3.43^BCb^	3.65 ± 0.032	315.26 ± 2.5^Bb^	2.97 ± 0.011^Bb^
	H1H3 (150)	11,061 ± 81.27^Aa^	399.1 ± 3.49^Aa^	3.62 ± 0.033	328.44 ± 2.54^Aa^	2.97 ± 0.011^Bb^
	H2H2 (78)	10,722 ± 104.5^Bbc^	383.32 ± 4.39^BCbc^	3.58 ± 0.042	316.19 ± 3.2^Bb^	2.95 ± 0.015^Bb^
	H2H3 (131)	10,717 ± 87.66^Bbc^	389.21 ± 3.74^ABb^	3.62 ± 0.036	317.87 ± 2.73^Bb^	2.96 ± 0.012^Bb^
	H3H3 (53)	10,884 ± 120.25^ab^	388.23 ± 4.99^b^	3.58 ± 0.048	329.01 ± 3.64^Aa^	3.02 ± 0.017^Aa^
	*P*	<.0001**	<.0001**	0.5127	<.0001**	0.0163*

*H* means haplotype; H1: CCCTC, H2: CCTTC, H3: TGCCT, and H4: CCCCT; ^*^ indicates *P* < 0.05; ^**^ indicates *P* < 0.01; ^a, b, c^ within the same column with different superscripts means *P* < 0.05; ^A, B, C^ within the same column with different superscripts means *P* < 0.01.

### Prediction of TFBSs variations in promoter region of *ATF3* gene

With regard to the four SNPs on the 5’promoter region of *ATF3* gene, we predicted the variation of TFBSs after mutation using JASPAR software. As the results shown in Table 6, the A allele in g.72834301C > A created the putative binding sites for the transcription factor E2F3 (E2F transcription factor 3; relative score = 0.88) and Zfp423 (zinc finger protein 423; relative score = 0.89). The binding sites for the transcription factor Bcl6 (B-cell CLL/lymphoma 6) was invented because of the A allele in g.72834229C > A (relative score = 0.87), and g.72833969A > G resulted in the appearing of the binding sites for transcription factor STAT3 (signal transducer and activator of transcription 3) after the substitution by G allele (relative score = 0.95). It was also indicated that the binding site for transcription factor Zfp423 was arisen due to the T allele in g.72833562G > T (relative score = 0.88).

**Table 6:** Transcription factor binding sites (TFBSs) prediction for *ATF3* gene

SNPs	Allele	TFBSs
g.72834301C > A	C	**-**
	A	E2F3, Zfp423
g.72834229C > A	C	-
	A	Bcl6
g.72833969A > G	A	-
	G	STAT3
g.72833562G > T	G	-
	T	Zfp423

- means no predicted TFBSs.

### Variant structure of CDKN1A protein caused by missense mutation

There was a missense mutation (p.Arg91Trp) on CDKN1A protein caused by c.271C > T. The results from the SOPMA SERVER revealed that α-helix was changed from 32.92% to 32.3%, β-turn from 4.35% to 5.59%, and random coil from 49.69% to 49.07% (Table 7), indicating that the CDKN1A protein secondary structure was altered from arginine to tryptophan.

**Table 7:** Alteration of CDKN1A protein caused by the mutation

SNPs	Allele	α-helix	Extended strand	β-turn	Random coil
c.271C > T	C	32.92	13.04	4.35	49.69
	T	32.3	13.04	5.59	49.07

## Discussion

This study was a follow-up investigation of our previous RNA-Seq work, in which the *ATF3* and *CDKN1A* genes were potentially associated with milk protein and fat percentage [9]. Here, we first determined that SNPs within the *ATF3* and *CDKN1A* genes were significantly associated with milk production traits in dairy cattle. Of these, four SNPs, g.72834301C > A, g.72834229C > A, g.72833969A > G, and g.72833562G > T, potentially changing the *ATF3* promoter activity, and one SNP, c.271C > T, potentially altering the CDKN1A protein secondary structure, might be potentially causal mutations.

With regard to the associations of *ATF3* and *CDKN1A* with five milk traits, we found that eight SNPs showed different associations between the first and second lactations (Tables 2 and 3). The possible reason may be that different number of cows were used for association analysis (1093 cows in the first lactation versus 769 cows in the second lactation) because 324 cows merely completed their milking of first lactation, which could impact the statistical significance. In addition, the physiologic status of cows between the first and second lactations are different as well, generally, cows show higher milk production in the second lactation. Further, the directions of allelic effects of the eight SNPs on the milk traits were almost consistent between the two lactations. Studies revealed that haplotype blocks in human genome is independent of their surrounding areas with regard to LD [15–17], and the haplotype analyses were widely applied to the genetic variation studies [18, 19]. Our haplotypes analyses showed that the SNPs were highly linked, and the haplotypes were also significantly associated with milk yield, fat yield, fat percentage, protein yield, and protein percentage, which were consistent with the associations of SNPs with milk traits.

*ATF3* is involved in the TLR4 (toll-like receptor 4) signaling pathway, and its expression was markedly increased causing by chemotaxis and diapedesis in vitro, indicating that *ATF3* acts as the early adaptive-response gene having an important role in maintaining the cellular homeostasis [20]. It was reported that *ATF3* activates the cyclin D1 expression, thereby stimulating the mouse hepatocellular proliferation [21]. As we know, liver plays a key role in lipid metabolism, including fatty acid uptake, synthesis and oxidation, glycerolipid synthesis, and triacylglycerol export [22]. Subsequently, Invernizzi et al. found *ATF3* may modulate milk fat synthesis during lactation by participating in the endoplasmic reticulum stress pathway [23, 24]. Our results also showed a significant relationship between SNP polymorphism of *ATF3* gene and fat yield, thus it can be seen that *ATF3* may be associated with lipid metabolism. The CDKN1A (p21) protein regulates the cell cycle at G1 and S phase by inhibiting the activity of cyclin-CDK2, −CDK1, and –CDK4/6 complexes [25]. Li and Capuco conducted a systematic search for estrogen-responsive genes in bovine mammary gland, and identified 23 regulatory networks, of these, *CDKN1A* gene occupied a focal position in the network that functions as cell cycle, cellular movement and cancers, indicating that *CDKN1A* may play an important role in mammary gland development [26]. Together, *ATF3* and *CDKN1A* were considered as important promising candidate genes for milk production traits.

As we know, SNPs in transcription factor binding sites could lead to allele-specific binding of transcription factors and enhancing or repressing the gene expression [27]. Studies have revealed that transcription factor E2F3 [28–31], Zfp423 [32, 33], and STAT3 [34, 35] could activate or repress the gene expression, and Bcl6 might play as a repressor for gene expression [36, 37]. Our results showed that the milk yield, fat yield, fat percentage, protein yield, and protein percentage were evidently decreased or had the downtrends in genotype AA, AA, and GG severally causing by g.72834301C > A, g.72834229C > A, and g.72833969A > G, indicating that the transcription factors E2F3, Zfp423, Bcl6, and STAT3, might prejudice the milk production by repressing the expression of *ATF3* gene. On the contrary, the transcription factor Zfp423 might be also beneficial to the milk production through activating the expression of *ATF3* gene, because that the genotype TT causing by g.72833562G > T increased the milk yield, fat yield, fat percentage, protein yield, and protein percentage in the two lactations. Overall, our findings suggested that the four SNPs, g.72834301C > A, g.72834229C > A, g.72833969A > G, and g.72833562G > T in *ATF3* gene, might be the potential mutations in milk production traits by changing promoter activity in Holstein cattle.

In the present, we found the SNP c.271C > T of *CDKN1A* gene was a missense mutation that caused an amino acid substitution from arginine to tryptophan, and the association analyses showed that this SNP was remarkably associated with milk yield and fat percentage in the first lactation, and milk yield, fat yield, protein yield, and protein percentage in the second lactation. Importantly, the changes of the protein structure causing by this amino acid substitution showed that the α-helix changed from 32.92% to 32.3%. Generally, the α-helix was preferably located at the core of the protein and had important functions in proteins for flexibility and conformational changes [38], and it was presumed that the CDKN1A protein might be more stable in conformation when the base was C. Hence, the SNP c.271C > T in *CDKN1A* gene might be another potential functional mutation for milk production through changing the protein structure in dairy cattle. Further in-depth investigation should be performed to validate the biological functions of these SNPs.

## Conclusions

In this study, we totally identified 19 and five SNPs in the *ATF3* and *CDKN1A* genes, respectively, and observed their associations with milk yield and milk composition. Of these, four SNPs in *ATF3* gene altered the specific TF binding sites thereby potentially changed promoter activities, and one SNP in *CDKN1A* gene changed the protein secondary structure, might be the potential causal mutations. In a word, our study first determined the significant genetic effects of *ATF3* and *CDKN1A* genes on milk yield and composition traits in dairy cattle and will be available for marker-assisted breeding based on further validation.

## Additional files

Additional file 1: Descriptive statistics of phenotypic values for dairy production traits in two lactations. (XLSX 9 kb)

Additional file 2: Primers and procedures for PCR or touch-down PCR used in SNPs identification of *ATF3* and *CDKN1A* genes. (XLSX 15 kb)

Additional file 3: Additive, dominant and allele substitution effects of 19 SNPs on milk production traits of *ATF3* gene in Chinese Holstein cattle during two lactations. (XLSX 17 kb)

Additional file 4: Additive, dominant and allele substitution effects of 5 SNPs on milk production traits of *CDKN1A* gene in Chinese Holstein cattle during two lactations. (XLSX 10 kb)

## Abbreviations

a: Additive effect
ATF3: Activating transcription factor 3
Bcl6: B-cell CLL/lymphoma 6
CDKN1A: Cyclin dependent kinase inhibitor 1A
CREB: cAMP responsive element-binding
d: Dominant effect
DHI: Dairy herd improvement
E2F3: E2F transcription factor 3
EBVs: Estimate breeding values
GWAS: Genome-wide association study
IPA: Ingenuity Pathway Analysis
LD: linkage disequilibrium
MALDI-TOFMS: Matrix-assisted laser desorption/ionization time of flight mass spectrometry
QTL: Quantitative trait locus
RNA-Seq: RNA sequencing
STAT3: Signal transducer and activator of transcription 3
TF: Transcription factor
TFBSs: Transcription factor binding sites
TLR4: Toll-like receptor 4
UTR: Un-translated region
Zfp423: Zinc finger protein 423
α: Substitution effect
